# A cross-sectional study on sleep length, quality, and mobile phone use among Moroccan adolescents

**DOI:** 10.11604/pamj.2022.41.252.25456

**Published:** 2022-03-28

**Authors:** Marouane Moustakbal, Souad Belabbes Maataoui

**Affiliations:** 1 Biochemistry, Neurosciences, Natural Resources and Environment Laboratory, Faculty of Sciences and Technology, Hassan First University, Settat, Morocco

**Keywords:** Adolescent, sleep length, sleep quality, mobile phone, Morocco

## Abstract

**Introduction:**

sleep plays an important role in learning and the formation of memories among adolescents, and lack of sleep affects their health, safety, and school performance, especially in the age of mobile phones. This study´s aim was to investigate sleep length and sleep quality among Moroccan adolescents and assess their relationship to mobile phone use.

**Methods:**

the population of this cross-sectional study included all the adolescent students aged 12-20 years, living in the Settat region, Morocco. We used proportionate stratified random sampling to select 800 participants from schools in the region. The researchers used a self-developed questionnaire coupled with the Pittsburgh Sleep Quality Index questionnaire to measure sleep length and evaluate sleep quality, respectively. We performed the statistical analysis using a combination of parametric and nonparametric tests to assess sleep length variables and PSQI score relationship to different independent variables.

**Results:**

participation rate was 91.2% (n = 730). The mean total sleep time was 8.01 hrs (SD = 1.46 hrs). Wake-up time was 8:17 a.m. (SD = 2.33 hrs), among late-night mobile phone users and 8:00 a.m. (SD = 2.11 hrs) among non-users. Overall, 76.7% of students from rural schools and 66.5% of students from urban schools reported poor sleep quality (p-value = 0.002). Most late-night mobile phone users (78%; p-value = 0.001) had poor sleep quality. Sleep quality decreased by age and in physically and psychologically unhealthy adolescents (p-value <0.001).

**Conclusion:**

a high percentage of adolescents in the Settat region suffer from poor sleep quality and sleep deprivation, especially among those who use cell phones before going to sleep. We advise parents and the Moroccan education system to promote good sleep habits and reduce the use of such devices in the bedroom by adolescents.

## Introduction

Adolescence is a period of physical, psychological, and social transition from childhood to adulthood [1]. Adolescent is a crucial phase in human life that requires extreme parental care, guidance, and empathy [2]. At present, there are about 7 million young people aged between 12 and 20 years in Morocco, which represent a very large and significant proportion of the country´s population [3]. Maintaining the physical and mental health of this important part of Morocco population will affect to a large degree the future economic development of the country. Therefore, protecting the youth from some unhealthy habits they may gain during adolescence is of vital importance. The type of attention that Moroccan adolescents usually receive, if they receive any, is too often focused on a specific behavioural problem such as childbearing, substance abuse or criminal involvement. However, there is an important health issue that could affect a larger proportion of Moroccan population in silence, which is the chronic sleep loss and poor sleep quality among adolescents. Chronic sleep loss in adolescence is an important public health issue [4]. This health problem is becoming increasingly recognized internationally as a significant concern, with many countries reporting high incidences of sleep disturbance among their youth [5].

The recommended duration of sleep for teens is 8-10 hours per night, but adolescents rarely attain this amount [6, 7]. In adolescence, insufficient sleep, inadequate sleep quality, and irregular sleep patterns are associated with impairment of cognitive capacities (memory, attention, learning, mood) and metabolic, immuno-inflammatory, and cardiovascular disorders [8]. Sleep deprivation is associated with daytime sleepiness, negative mood, increased likelihood of stimulant use, higher level of risk-taking behaviours, poor school performance and increased risk of unintentional injuries [9]. Further, mobile phones have become an important tool in daily life, especially among children and teens, as they are more inclined to embrace and use new technologies for communication and entertainment. However, some adolescents become so dependent on their phones that they take them even to bed before sleep. Several studies associated the massive use of phones among pre-teens and teenagers with high rates of sleep disturbances [10-12].

In Morocco and other North African countries, there is growing concern that insufficient sleep may take a toll on students´ health and well-being. Some parents and clinicians think that the emergence of new technologies has aggrieved this issue among the youth. Investigation in this area is imperative, as mobile phone use has infiltrated almost every aspect of adolescents’ daily life. Development of adequate responses and strategies to prevent sleep disturbances or restore altered sleep patterns is of major importance, but limited availability of the data regarding sleep duration, quality, and its association with mobile phone use in the Moroccan population is an obstacle to any meaningful interventions. Although some previous studies have described Moroccan students´ sleep duration, however to the best of our knowledge, no prior studies have focused on different sleep parameters or tested their relation to demographic characteristics or phone use. Therefore, the first objective of the present study is to determine the prevalence of poor sleep quality and reduced sleep length among Moroccan adolescents. The second objective is to examine the associations between adolescents’ sleep length, quality, and their individual demographic characteristics. The final objective of this study is to assess the relationship between different sleep parameters and the use of mobile phones in bed.

## Methods

**Study design:** we carried out a school-based, observational cross-sectional study. The choice of this design is partially due to the lack of financial resources and the absence of adequate sleep research centres necessary for experimental design and the study of behavioural effects on sleep deprivation. However, the most important advantage of cross-sectional studies is that they are quick and cheap. As there is no follow-up, fewer resources are required to run the study. With this design, we can collect data all at once and study multiple outcomes at the same time. Cross-sectional studies are the best way to determine prevalence and are useful for identifying associations that can then be more rigorously studied, using a cohort study or randomized controlled study.

**Setting:** we conducted the survey among school-attending adolescents in 2019 in Settat province, in the North center of Morocco. The region is a fertile agricultural plain with few urban centres. According to the 2014 census, the total population of the province was estimated to be 634,188 inhabitants in 2014, and the inhabitants of rural environment were 417,357, then the urbanization rate was 34.19%.

**Study population:** we based our sampling frame on the yearly updated database of middle and high school students, which was gained from the Settat regional directorate of education. The database included the total population of middle and high school students in the Settat region, which at the moment of the study was (N) = 23755 students. The database also included specific statistics dividing students by sex, age, school location, and school level. All schools comprised both genders and a mixture of day and boarding students. Generally, the students from the urban cities are the most affected by recent lifestyle changes occurring among youth, but with the onset of the digital age, the difference between urban and rural students has become less apparent. Thus, it was decided that participants from rural areas should be included too in the study population.

**Sampling:** using G-Power software (version 3.1) we calculated a priori that the sample size needed to perform t-test statistical comparisons with statistical analysis power of 0.80 and a small effect size (0.2) will be around 784 students. The population database regrouped all students in the region into two subgroups: students in urban schools and students in rural schools. Inclusion criteria comprised students from both middle and high schools, aged between 12 and 20 years, and who agreed to take part in the study. Based on these database statistics, we used the proportionate stratified sampling design to include participants based on their proportional allocation in the actual population. A stratified sample can be smaller than simple random samples, which can save a lot of time, money, and effort for the researchers. This type of sampling technique has a high statistical precision compared to simple random sampling and guarantees better coverage of the population and less selection bias. The cumulative population per stratum was divided by the total population and multiplied by the estimated sample size to determine the number of students that would be required to take part per stratum. Based on this calculation, seven urban and two rural schools were selected by probability proportional to size. In strata 1, we included 7 randomly selected urban schools. In strata 2, we included 2 randomly selected rural schools. Then, we randomly drew 800 adolescents from student´s lists of the 9 selected schools to take part in the study.

**Procedures:** we developed the tool in Arabic, the students´ native language, which included three questionnaires that were pretested prior to data collection. It included a questionnaire about demographic variables such as age, sex, area of residence, school level, parent´s job, physical activity and health status. In addition, we measured the weights and heights of the adolescents to calculate the body mass index (BMI) and determine their nutritional status. Also, the questionnaire included questions about mobile phone use before sleep and its duration. The second questionnaires included questions about sleep length characteristics, such as usual bedtime, sleep latency, wake-up time, and total sleep duration.

Finally, we used the Arabic version of the Pittsburgh Sleep Quality Index (PSQI) questionnaire, which comprises 19 self-rated questions. The 19 self-rated questions assess a wide variety of factors relating to sleep quality, as well as frequency and severity of specific sleep-related issues [13]. The PSQI reliability in this study was acceptable (Cronbach´s alpha = 0.79). The sampling required an average of one week for each school. We travelled to every selected school between the period of November 2019 and January 2020, distributed the questionnaires among selected participants and asked the students to complete them anonymously in the classrooms. They filled out the forms in our presence in order to assist students and answer questions. The Instrument took about 30 minutes to complete.

**Study variables:** the independent variables of the study were the following: age, sex, area of residence, BMI, school level, family socioeconomic status, physical activity, physical health status, psychological well-being, mobile phone use before sleep, and duration of mobile phone use. We split socioeconomic status into three groups: low, middle, and high-income families. We converted two continuous independent variables (age, BMI) to categorical variable with three outcomes, respectively. Furthermore, we devised health status among different participants in two different variables, one variable reflected on physical health impairment and the second one measured psychological well-being. Participant with one of the underlying conditions: hypertension, chronic cough, asthma, cardiopathy, diabetes, chronic pain, chronic dermatitis and epileptic seizures, were deemed to be physically unhealthy. Measure of psychological well-being derived from respondents´ self-reported depression and anxiety, we deemed any participant with one or both conditions as having negative psychological well-being.

We divided the dependent variables into two categories: the first category comprised four continuous parameters related to sleep length: the usual bedtime; sleep latency in minutes; wake-up time; and total sleep in hours. The second category comprised one categorical variable related to perceived sleep quality, that we calculated using the Pittsburgh Sleep Quality Index (PSQI). We regrouped the 19 items into seven component scores, each weighted equally on a scale ranging from 0 to 3. We calculated the global PSQI score by the summation of the seven component scores, which has ranges from 0 to 21 with higher scores showing worse sleep quality [13]. The variable has only two outcomes: a global score equal to or greater than 5 indicates poor sleep quality, while a score less than 5 indicates good sleep quality [13].

**Data analysis:** we presented descriptive statistics for personal characteristics of the adolescents who took part in the study using frequencies and percentages, while we described our numerical parameters with mean and standard deviations. We conducted the Shapiro-Wilk test to examine the normality of the continuous variables. Pairwise deletion was used to address missing data. We tested the associations between independent categorical variables and continuous parameters related to sleep length. Subsequently, we performed Student´s t-test (normally distributed data) or the Mann-Whitney U test (non-normally distributed data) to compare the sleep length parameters and different dichotomous independent variables. For association between independent variables with over two levels and continuous parameters related to sleep length, Kruskal-Wallis test was used to assess their relationship.

Cross-tabulations (contingency tables) were used to examine the relationship between independent categorical variables and sleep quality variable. For cross-tabs with independent nominal variables, we used the Cramer's V test (the normalized version of the χ^2^ test statistic) to assess their relationship to the sleep quality variable. Whereas, for cross-tabs with independent ordinal variables, we used the Goodman and Kruskal´s gamma to assess their relationship to the sleep quality variable. Finally, we tested the statistical relationship between sleep quality as the dependent variable and sleep length and duration of mobile phone use as independent variables, using Student´s t-test for normally distributed data and the Mann-Whitney U test for non-normally distributed data. We analysed all the data using IBM SPSS (IBM Corp. 2012. IBM SPSS Statistics for Windows, version 21.0. NY, EUA). We considered results with a p-value ≤ 0.05 as statistically significant.

**Ethical considerations:** we obtained ethical clearance from the Settat regional directorate of education and from parents´ association in every school visited. Informed consent was obtained from the students verbally and written in every administered questionnaire. We included no personal identifiers in the questionnaire. Anonymity and confidentiality were maintained throughout data collection, storage, and analysis.

**Funding statement:** this research did not receive any specific grant from funding agencies in the public, commercial, or not-for-profit sectors.

## Results

Seven hundred and thirty students completed the three questionnaires (44.5% males and 55.5% females). The mean age (±SD) was 15.64 ± 1.66 years and the mean BMI was 20.17 ± 2.50 kg/m2. The mean total sleep time was 8.01 hrs (SD = 1.46 hrs). We regrouped the general characteristics of the adolescents who took part in the study in Table 1. In Table 2, we represent participants´ bedtimes, Sleep latency, wake-up times, and total sleep durations within different socio-demographic groups (mean ± SD).

**Table 1:** general characteristics of the adolescents who took part in the study

Variables	Count	(%)	Missing Data
**Sex**			
Male	325	44.5	0
Female	405	55.5	
**Age**			
12 - 14	223	30.5	0
15 - 17	404	55.3	
18 - 20	103	14.1	
**Area of residence**			
Rural	390	53.4	0
Urban	340	46.6	
**BMI**			
Underweight	151	25.9	147
Normal	416	71.3	
Overweight	16	2.7	
**School level**			
Middle School	343	46.9	0
High school	387	53.1	
**Family socio-economic Status**			
Low income	616	86.4	17
Middle Income	79	11.1	
High Income	18	2.5	
**Mobile phone use before sleep**			
Yes	337	46.2	1
No	392	53.8	
**Physical activity**			
Yes	323	44.4	21
No	404	55.6	
**Physical health**			
Healthy	527	74.3	21
Unhealthy	182	25.7	
**Psychological well-being**			
Positive	397	55.2	11
Negative	322	44.8	

**Table 2:** bedtimes, sleep latency, wake-up times and total sleep durations within different socio-demographic groups (mean ± SD)

Variables	The usual bedtime			Sleep latency in minutes			Wake-up time			Total sleep in hours		
	Mean (p.m.) ±SD		p	Mean ±SD		p	Mean (p.m.) ±SD		p	Mean ±SD		p
**Area of residence**												
Rural	10:30	±1:16	<0.001^a^	0:33	±0:25	<0.001^b^	7:09	±01:12	0.009a	7:59	±1:41	0.623^a^
Urban	11:15	±1:18		0:29	±0:25		7:51	±01:12		8:03	±1:51	
**School level**												
Middle School	10:51	±1:20	0.945^a^	0:32	±0:26	0.917^b^	7:38	±01:18	0.001^a^	8:13	±1:52	0.004^a^
High school	10:50	±1:20		0:31	±0:23		7:19	±01:11		7:50	±1:39	
**Family Socio-economic Status**												
Low income	10:47	±1:21	0.018^c^	0:32	±0:25	0.012^c^	7:25	±01:16	0.002^c^	8:00	±1:46	0.748^c^
Middle Income	11:09	±1:13		0:26	±0:23		7:49	±01:05		8:10	±1:44	
High Income	11:13	±1:09		0:46	±0:30		7:50	±01:10		7:43	±1:57	
**Mobile phone use before sleep**												
Yes	11:15	±1:22	<0.001^a^	0:34	±0:25	0.002^b^	7:31	±01:14	0.407^a^	7:37	±1:50	<0.001^a^
No	10:29	±1:11		0:29	±0:25		7:26	±01:16		8:22	±1:38	
**BMI**												
Underweight	10:38	±1:17	0.02^c^	0:29	±0:23	0.437^c^	7:27	±01:16	0.730^c^	8:13	±1:41	0.183^c^
Normal	10:51	±1:19		0:32	±0:25		7:25	±01:16		7:57	±1:47	
Overweight	11:28	±0:56		0:41	±0:33		7:47	±01:23		7:35	±1:43	
**Physical health**												
Healthy	10:49	±1:18	0.303^a^	0:31	±0:25	0.520^b^	7:31	±01:13	0.054^a^	8:04	±1:44	0.144^a^
Unhealthy	10:56	±1:27		0:33	±0:26		7:18	±01:20		7:51	±1:50	
**Psychological well-being**												
Positive	10:44	±1:15	0.01^a^	0:27	±0:23	<0.001^b^	7:34	±01:15	0.033^a^	8:18	±1:41	<0.001^a^
Negative	10:59	±1:25		0:37	±0:26		7:22	±01:15		7:39	±1:49	

Note. BMI: body mass index, ^a^P-Value by t-test, ^b^P-Value by Mann-Whitney's U test, ^c^P-Value by Kruskal-Wallis test.

Sleep latency was 34 min (SD = 25 min) for mobile phone users and 29 min (SD = 25 min) for non-users. Mobile phone users had significantly higher sleep latency than non-users (p-value <0.01). There was no significant difference between the two groups in wake-up time, as those who used a mobile phone before sleep had a mean wake-up time of 7:31 a.m. (SD = 74 min) whereas non-users had a mean wake-time of 7: 26 a.m. (SD = 76 min; p-value = 0.407).

The total sleep time was significantly different between the two groups, the mobile phone users slept less than non-users. The mobile phone users had a total sleep time of 7.37 hrs (SD = 1.50 hrs), and the non-users had a total sleep time of 8.22 hrs (SD = 1.38 hrs; p-value <0.001). We measured the body mass index (BMI) in the population study. 25.9% of participants were underweight and had a total sleep time of 8.13 hrs (SD = 1.41 hrs), 71.4% were normal and had a total sleep time of 7.57 hrs (SD = 1.47 hrs), and 2.7% were overweight and had a total sleep time of 7.35 hrs (SD = 1.43 hrs). There was no significant difference in total sleep time between different groups (p-value = 0.183).

The differences between students from urban and rural areas were significant for bedtime, sleep latency, and wake-up time, but there was no significant difference in total sleep time. The students from urban areas had a mean bedtime of 11:15 p.m. (SD = 77 min), whereas students from rural areas had a mean bedtime of 10: 30 p.m. (SD = 76 min; p-value <0.001). The sleep latency in urban students was 30 min (SD = 25 min) and 33 min (SD = 25 min) in rural students (p-value = 0.009). The wake-up time for urban students was 7: 51 a.m. (SD = 72 min) and 7: 08 a.m. (SD = 73 min) for rural students (p-value <0.001). The urban students had a total sleep time of 8.02 hrs (SD = 1.52 hrs), and 7.59 hrs (SD = 1.42 hrs) for rural students (p-value = 0.623).

High school adolescents had an earlier wake-up time and a less total sleep time compared to middle school adolescents. The mean wake-up time of high school participants was 7:19 a.m. (SD = 71 min) and 7:39 a.m. (SD = 79 min) for middle school children (p-value =0.001). The total sleep time for high school students was 7.50 hrs (SD = 1.40 hrs), whereas 8.12 hrs (SD = 1.53 hrs) was the total sleep time for middle school students (p-value = 0.004). Participants from different socioeconomic statuses had significant differences in bedtime, sleep latency, and wake-up time, whereas there were no differences in total sleep time between various groups. We summarized the results in Table 2 with significance levels.

Overall, the physically unhealthy students with at least one chronic impairment were about 25.7% (n=182) while physically healthy students were about 74.3% (n=527). We observed that physically unhealthy adolescents were not significantly different from healthy adolescents regarding bedtime, sleep latency, wake-up time, or total sleep time. Negative psychological well-being was important among participants, 44.8% (n=322) of participants suggested surfing from anxiety or depression and only 55.2% (n=397) had positive psychological well-being. We observed significant differences in all four sleep length measures between psychologically healthy and unhealthy students, where bedtime in healthy individuals was 10: 44 p.m. (SD = 75 min) and 10: 59 p.m. (SD = 85 min; p-value <0.009). Sleep latency in healthy and unhealthy students was 27 min (SD = 23 min) and 37 min (SD = 26 min) respectively (p-value <0.001). Psychologically healthy adolescents had a wake-up time of 7: 34 a.m. (SD = 75 min) and 7: 22 a.m. (SD = 75 min) for unhealthy adolescents (p-value =0.034). Regarding the total sleep time, it was about 8.18 hrs (SD = 1.41 hrs) in psychologically healthy individuals and 7.39 hrs (SD = 1.49 hrs) in psychologically unhealthy individuals, the adolescents with positive psychological well-being had more sleep time compared to individuals with negative psychological well-being (p-value <0.001).

Regarding sleep quality, Table 3 summarizes the associations between PSQI results and different Socio-Demographic Characteristics. Overall, 28.1% participants had a good sleep quality, whereas 71.9% participants had poor sleep quality. The proportion of sleep quality was similar between individuals from different sexes (p-value = 0.433). Adolescents from different age categories had different sleep qualities, as 58% of young adolescents aged 12 to 14 had a poor sleep quality, compared to 76% in middle adolescents aged 15 to 17 and 84% in late adolescents. We observed that older adolescents have poorer sleep quality compared to their younger counterparts (p-value <0.001). A considerable proportion of students from rural areas reported poor sleep quality (77%) compared to urban students who reported less poor sleep quality (66%). Students from rural areas had poorer sleep quality than the urban students (p-value = 0.002). Around 78% of high school adolescents had a poor sleep quality, compared to 65% reported by middle school adolescents (p-value <0.001).

**Table 3:** associations between sleep quality and socio-demographic characteristics

	Overall Sleep Quality (PSQI) N=730				
**Variables**	**Good sleep quality a**		**Poor sleep quality b**		**p**
	**Count**	**(%)**	**Count**	**(%)**	
**Sex**					
Male	96	(30)	229	(70)	0.433^c^
Female	109	(27)	296	(73)	
**Age**					
12 - 14	93	(42)	130	(58)	<0.001^d^
15 - 17	96	(24)	308	(76)	
18 - 20	16	(16)	87	(84)	
**Area of residence**					
Rural	91	(23)	299	(77)	0.002^c^
Urban	114	(34)	226	(66)	
**BMI**					
Underweight	49	(32)	102	(68)	0.084^d^
Normal	107	(26)	309	(74)	
Overweight	3	(19)	13	(81)	
**School level**					
Middle School	119	(35)	224	(65)	<0.001^c^
High school	86	(22)	301	(78)	
**Family Socio-economic Status**					
Low income	169	(27)	447	(73)	0.318^d^
Middle Income	29	(37)	50	(63)	
High Income	3	(17)	15	(83)	
**Mobile phone use before sleep**					
Yes	74	(22)	263	(78)	0.001^c^
No	131	(33)	261	(67)	
**Physical activity**					
Yes	18	(24)	58	(76)	0.363^c^
No	187	(29)	466	(71)	
**Physical health**					
Healthy	171	(32)	356	(68)	<0.001^c^
Unhealthy	26	(14)	156	(86)	
**Psychological well-being**					
Positive	156	(39)	241	(61)	<0.001^c^
Negative	43	(13)	279	(87)	

Note. PSQI: Pittsburgh Sleep Quality Index, BMI: body mass index, ^a^Good sleep quality: PSQI score <5.0, ^b^ Poor sleep quality: PSQI score ≥5.0, ^c^P-Value by Cramer's V test, ^d^P-Value by Gamma test

Adolescents using a mobile phone before going to sleep were more likely to have a lower sleep quality compared to those not using mobile phones before going to sleep (p-value = 0.001), with 78% and 67% reporting poor sleep quality, respectively. Participants with physical health problems reported in 86% of cases having poor sleep quality compared to 68% in healthy participants, showing that physically healthy individuals have a better sleep quality than their unhealthy counterparts (p-value <0.001). The most noticeable differences came from psychological well-being, with adolescents suffering from negative psychological well-being reporting 87% of poor sleep quality, compared to 61% in the group with positive psychological well-being (p-value <0.001).

We found no clear associations between BMI, Family Socioeconomic Status, Physical Activity, and sleep quality among adolescents (p-value =0.084; 0.318; 0.363, respectively). We presented the associations between sleep quality, bedtime, wake-up time, sleep latency, total sleep time, and also the duration of mobile phone use in Table 4. Students with poor sleep quality were sleep-deprived with only 7: 41 hrs (SD = 1.49 hrs) total sleep time, 72 minutes (SD = 7 min) less than their counterparts with good sleep quality, who had 8.53 hrs (SD = 1.16 hrs) total sleep time (p-value <0.001). The adolescents with poor sleep quality used mobile phones more by about 70 minutes (SD = 18 min) compared to adolescents with good sleep quality (p-value <0.001).

**Table 4:** association between sleep quality, sleep length and duration of mobile phone use

Variables	Overall Sleep Quality (PSQI) N=730				
	Good sleep quality a (n=205)		Poor sleep quality b (n=525)		p
	Mean	±SD	Mean	±SD	
**Bedtime** (p.m. ±SD hours)	10:27	±1:08	11:00	±1:22	<0.001^c^
**Sleep latency** (minutes ±SD)	0:16	±0:15	0:37	±0:26	<0.001^d^
**Wake up time** (a.m. ±SD hours)	07:38	±1:12	7:25	±1:16	0.03^c^
**Total sleep time** (hours ±SD)	8:53	±1:16	7:41	±1:49	<0.001^c^
**Duration of mobile phone use** (hours ±SD)	4:08	±3:35	5:18	±3:55	<0.001^d^

Note. PSQI: Pittsburgh Sleep Quality Index, ^a^Good sleep quality: PSQI score <5.0, ^b^Poor sleep quality: PSQI score ≥5.0, ^c^P-Value by t-test, ^d^P-Value by Mann-Whitney's U test.

## Discussion

The primary aim of this study was to determine the prevalence of sleep disturbances among school attending adolescents in a North African context. About 44% of adolescents reported suffering from sleep deprivation, as they sleep less than the 8-hour minimum recommended for their age [5, 14-16]. Overall, 72% of adolescent students reported having poor quality sleep. The secondary aim was to assess the relationship between different sleep parameters and demographic characteristics. Adolescent´s age was significantly associated with sleep quality; as poor sleep quality was higher among older participants than younger ones. In addition, our results confirmed that poor sleep quality was related to decreased levels of physical and psychological well-being, respectively. Finally, the present study evaluated the association between sleep length, sleep quality, and the use of mobile phones before bedtime among adolescents. Forty-six percent (46%) of participants reported using mobile phones at night, before going to sleep. Nighttime use of mobile phones was associated with higher sleep deprivation, as the adolescents using phone before sleep, slept 45 minutes (SD = 8 min; p-value <0.001) less than non-users. In addition, sleep quality was also associated with the use of mobile phones before sleep, as poor sleep quality was higher among mobile phone users than non-users.

The mean total sleep time in this study was 8.01 hrs (1.46), which is in line with the minimum recommended sleep duration for adolescents [5]. Nevertheless, more than a third (44%) of participants reported having sleep deprivation. This result aligns with the global pattern of insufficient adolescent sleep, as revealed in a study by Sharman and Illingworth [17]. In fact, adolescents today are more likely to experience insufficient sleep than they did 20 years ago [17]. This result is greater than those found in a 2015 study about obesity among Moroccan adolescents, where approximately 37% of adolescents had a total sleep time less than 8 hours [18]. But we should take it into consideration that the Moroccan government had recently implemented permanently a daylight-saving time (DST), changing time from GMT to GMT+1 [19, 20]. This recent change may have influenced the results in this study, because of the negative effects of DST onset on sleep, as documented in a previous study by Medina and Ebben on high school adolescents [21]. All the schools included in the study had a morning start time at 8: 30 a.m., which is in line with the recommendations of the American Academy of Pediatrics [22]. But the permanent implementation of DST makes the real morning start time at 7:30 a.m. Evidence strongly implicates earlier school start times (i.e., before 8:30 a.m.) as a key contributor to insufficient sleep, as well as circadian rhythm disruption, among adolescents [23-25].

Our results suggested that poor quality sleep is highly prevalent in participants, as about 72% of students reported to have poor sleep quality. Results from a study on adolescents from similar cultural backgrounds in Turkey, reported that 66.6% of participants were found to have poor sleep quality [26]. Similarly, a study from Beirut has found that 76.5% of the Lebanese high school students were not satisfied with their sleep quality [27]. The above results indicate that the poor sleep quality rates among adolescents in developing countries are similar, as reported in a review study about sleep quality in low and middle-income countries [28]. Notably, Sleep quality among our participants declined with increasing age. This contradicts a previous study on Nigerian adolescents that found no significant effect of age on sleep quality [29]. A study on Iranian adolescents had found significant differences in sleep quality among adolescents from different ages [30].

In addition, this study results confirmed the relation between poor-quality sleep and reported physical clinical complaints. Auvinen suggested in his study that poor-sleep quality is a risk factor for neck, shoulder and low back pains in adolescence [31]. Moreover, Sleep intervenes in a significant number of clinical complaints, including headache and different chronic pains [9]. This may be ascribed to less effective rest from their sleep (due to the shortage of sleep and the poor sleep quality), and subsequently development of health problems, such as chronic pain and fatigue. However, the results of the present study cannot infer a causality between sleep and health effects, because of the study adopted observational design.

The effect of psychological well-being on overall sleep quality was the most noticeable among the participants, with most adolescents with negative psychological well-being expressing a poor quality of sleep. Many studies have shown that there is a bidirectional association between sleep quality and anxiety symptoms, where sleep quality can be a secondary symptom to psychiatric complaints [32, 33]. These findings are consistent with previous studies linking perceived stress with poor sleep quality among adolescents [34, 35]. A systematic review of 9 studies suggested that there is a vicious cycle between poor sleep and many psychological disorders, with reports of reciprocal or bidirectional relationships between them [36].

When considering the use of mobile phones at bedtime or during the night, our study has found that about 46% of adolescents reported using phones at night before going to sleep, which is comparable to what Calamaro found in a study on adolescents from the suburb of Philadelphia, where 44% of the subjects reported talking on the telephone after 9 pm [11]. Other studies on adolescents in other regions of the world have found a similar result [37, 38]. One explanation of excessive smartphone using may be the frequent visiting of social media sites, as for virtually all young people, it is the way they connect and communicate in this digital age. Our findings have shown a significant reduction in sleep among adolescents who used mobile phones before sleep, with 45 minutes (SD = 8 min) less sleep than their counterparts not using mobile phones before sleep. This finding is in line with studies on mobile phone use after lights out in Japanese adolescents, that significantly associated sleep length with mobile phone use among adolescents [12, 39, 40]. Cain and Gradisar hypothesized that the use of mobile phone in bed can displace sleep time and other activities associated with good sleep hygiene, and thus results in later bedtimes and shorter sleep duration [10].

In addition, our study found that there is a negative relationship between mobile phone use and sleep quality, as adolescent using mobile phone before sleep reported a significant decrease in their overall sleep quality compared to non-users. These results are in agreement with previous studies revealing the relationship between smartphone use severity and sleep quality: according to Liu and all, Chinese adolescents with high level of mobile phone addiction were predicted to have low sleep quality [41]. Prior to this study, Munezawa reported the same results in a study on Japanese adolescents in a nationwide cross-sectional survey, suggesting that mobile phone use may play an important role in sleep problems among adolescents worldwide [12]. Poor sleep quality may arise from the use of interactive technological activities (particularly social media) due to increased mental stimulation and temporal displacement, compared to more passive activities such as watching television [42]. Another explanation for these results may be the effect of exposure to light, especially short wavelength light emitted by phones, that can counteract the machinery in the pineal gland responsible for producing melatonin, a hormone that helps control the sleep-wake cycle [43].

Several limitations of the present study should be noted. A major limitation of this study was that this is a cross-sectional study, which may be more susceptible to recall bias, and it is not possible to make associations of cause and effect from the results. Future studies may conduct longitudinal research or experimental research to confirm the causal relationships among variables. Another key limitation is the fact both sleep parameters and phone use habits were evaluated according to the statements of the students, rather than according to reports taken from a more objective source. Therefore, future research may try to collect data from multiple informants (e.g. parents, teachers, and peers). In spite of these limitations, we believe that the results of the study are important to raise awareness, considering that in Morocco there are few studies that have been conducted on this subject. We believe that the use of proportionate sampling gives this study´s results robust generalizability among Moroccan adolescents.

## Conclusion

Sleep plays an essential role in physical, emotional and behavior health of adolescents. Understanding their sleep patterns and sleep duration requirements remains essential to provide adequate recommendations. Our observations provide an important outlook on sleep characteristics among Moroccan adolescents, opening the door to more experimental interventions in the future. First, one of the more obvious targets for intervention is to realign the school day to the adolescent biological clock through delaying school start times. Considering the permanently implemented daylight-saving time in Morocco, no school should start before 09: 30 a.m., as recommended by the American Academy of Pediatrics. Second, parents and educators need to help adolescents learn that mobile phone addiction has adverse effects on sleep quality, and should assist them in reducing the dependence on mobile phones. Finally, parents should set an appropriate bedtime, regulate screen use, as well as maintain bedrooms as a screen free zone.

### What is known about this topic

- Sleep is vital for people of any age;
- Adolescents through profound mental, physical, social, and emotional development require quality sleep;
- Mobile phones have become an integral part of adolescent culture;

### What this study adds

- Adolescents in the Settat region - Morocco, suffers from sleep deprivation;
- Poor sleep quality exists among the high percentage of schooling adolescents in Settat, Morocco;
- This study negatively associated both sleep length and quality with the use of mobile phones.
